# The development, validation, and *in vivo* testing of a high-precision bronchial epithelial lining fluid sampling device

**DOI:** 10.3389/fmed.2023.1172622

**Published:** 2023-07-26

**Authors:** Akash Gupta, Janette K. Burgess, Dirk-Jan Slebos, Simon D. Pouwels

**Affiliations:** ^1^Department of Pulmonology, University Medical Center Groningen, University of Groningen, Groningen, Netherlands; ^2^Department of Pathology and Medical Biology, University Medical Center Groningen, University of Groningen, Groningen, Netherlands; ^3^University Medical Center Groningen, Groningen Research Institute for Asthma and COPD (GRIAC), University of Groningen, Groningen, Netherlands; ^4^University Medical Center Groningen, W.J. Kolff Institute for Biomedical Engineering and Materials Science-FB41, University of Groningen, Groningen, Netherlands

**Keywords:** airway sampling, COPD, epithelial lining fluid, bronchial wash, biomarkers

## Abstract

**Introduction:**

Analysis of respiratory biomarkers or pharmaceutical drug concentrations in bronchial epithelial lining fluid (bELF) using a high-precision sampling method is crucial for effective clinical respiratory diagnostics and research. Here, we utilized a cellulose matrix as an absorptive probe for bELF sampling, subsequently testing the design of a device and sampling technique *in vivo*.

**Methods:**

The absorptive matrix [Whatman® qualitative filter paper (Grade *CF*-12)] was first tested through tissue-contact experiments on porcine airway tissue. The absorption and elution capacity of the matrix, as well as the laboratory processing and analysis method, was validated with a range of Interleukin-8 (CXCL8) and C-Reactive protein (CRP) stock solutions. Subsequently, the device’s design was optimized for universal in-house production and both, safe and efficient sampling. The airway sampling method was then tested in a group of 10 patients with Chronic Obstructive Pulmonary Disease (COPD). For each patient, a bELF sample was obtained using the newly developed bELF probe, as well as a reference 20 mL saline bronchial wash sample. Supernatants were assessed, using an immunoassay, for levels of the pro-inflammatory markers CXCL8, Myeloperoxidase (MPO), and CRP. The bELF samples were compared to bronchial wash.

**Results:**

The Whatman® qualitative filter paper (Grade *CF*-12) bELF probes adhered to porcine airway tissue, softening slightly upon wetting. The material maintained architectural integrity following the removal of the probes, leaving no residual fibers on the porcine airway mucosa. The bELF probe design was optimized for bronchoscopic delivery and in-house production. On average, a fully saturated bELF probe carried 32 μL of protein-rich fluid. The mean return of CXCL8 and CRP from samples collected from a serial dilution series (1, 5, 10, 20 ng/mL) was 69% (range 48%–87%). The bELF probe detected, on average, 7 (MPO), 14 (CRP), and 59 (CXCL8) times higher equivalent inflammatory protein concentrations in the collected bELF probe samples compared to the bronchial wash.

**Conclusion:**

The bELF probe is an effective absorptive technology for high-precision bELF sampling without dilution. With a simple in-house production procedure and bronchoscopic sampling technique, this method can be introduced in any bronchoscopic center for a consistent sampling of bELF.

## 1. Introduction

Advancements in clinical respiratory research and diagnostics are leading a shift toward high-throughput multi-omics approaches for profiling heterogeneous lung pathologies (1–4). With this shift, precision in sampling techniques has become increasingly important. Bronchial epithelial lining fluid (bELF) is a critical respiratory specimen for insights into fundamental pathobiological mechanisms of respiratory disease, establishing diagnoses, evaluating prognoses, and monitoring therapy. Constituents of epithelial lining fluid include suspended respiratory and immune cells, microorganisms, toxins, allergens, inhaled medications, and cellular secretory products (e.g., cytokines and growth factors) (5, 6). The conventional methods for sampling bELF, such as bronchial wash, depend on bronchoscopic saline lavage of a target bronchial branch. The sensitivity to detect biomolecules with low abundance is limited due to the extensive dilution of bELF and the absence of a standardized correction method (7). Additionally, the spatial informativity of the identified markers in the collected specimen is limited due to a lack of delineation of the surface area sampled and indiscriminate collection of bELF from downstream sites not visible under direct vision (3, 8).

An effective alternative to saline lavage is the application of a sampling probe, developed from a synthetic or cellulose-based absorptive matrix (9, 10), onto the mucosal layer of the target bronchial wall, collecting bELF through assimilation. The sampling probe collects bELF with no dilution, and sampling is limited to the contact area of the bronchial surface with the probe. These features improve the sensitivity and spatial informativity for detecting biomolecules in bELF.

Olympus first developed the bronchoscopic microsampling (BMS) method and commercialized a synthetic hydroxylated polyester (FHPE) and a cellulose-based absorptive tip into catheter systems for bELF sampling (9). Recently, a respiratory medicine group at Imperial College, together with Hunt Developments (UK) Ltd., commercialized the bronchosorption device, a soft synthetic absorptive tip with a similar catheter system. However, there is still scope for developing additional high-precision bELF sampling device approaches.

In this study, we set out to develop and investigate a novel bELF sampling probe with a high capacity of bELF absorption and reliable output while being simple and inexpensive to produce.

## 2. Materials and methods

### 2.1. Testing of absorptive materials

Three absorptive matrices were tested, the Whatman® Grade GF/C Glass microfiber filters (WHA1822090), Whatman® Grade 3 qualitative filter paper (WHA1003185), and Whatman® Grade *CF*-12 qualitative filter paper (WHA10535097; all from Cytiva, Massachusetts, United States).

### 2.2. bELF probe production and preparation for testing and patient sampling

**Figure 1:**
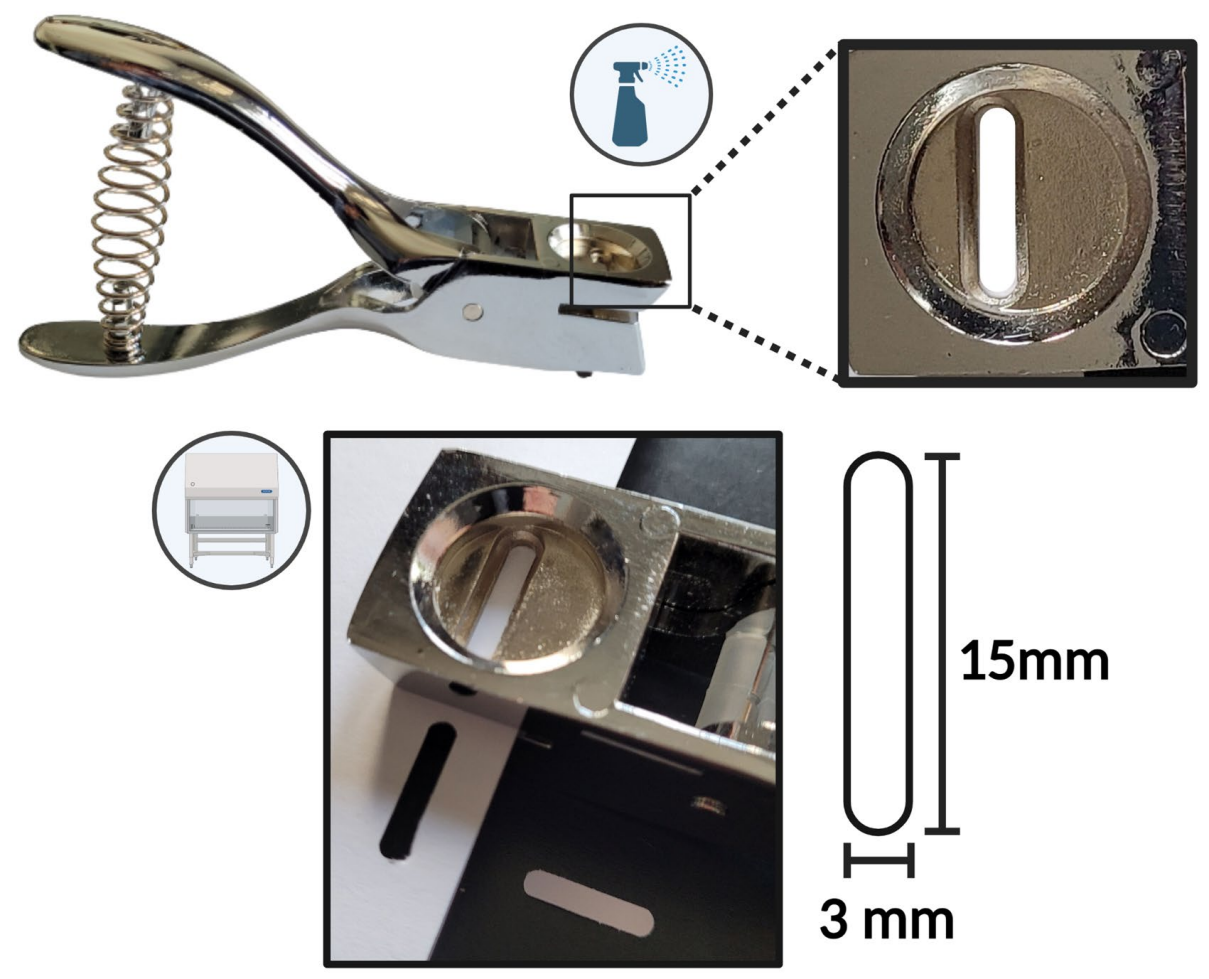
Schematic depicting the bronchial epithelial lining fluid (bELF) probe production tools and procedure.

The probes were produced in a clean working environment within a laminar flow cabinet. Probes were cut from a Whatman® qualitative filter paper (Grade *CF*-12) A4 sheet, Whatman® Glass microfiber filters (Grade GF/C) circles (⌀ 23 mm), Whatman® qualitative filter paper (Grade 3) circles (⌀ 23 mm), using an ethanol-sterilized hole puncher (Ninghai Haibei Stationery Co., Zhejiang, China), producing near-identical oval probes with dimensions of 15x3mm (Figure 1). To prepare for the dilution correction after sample collection, weight measurements of the empty tube and the dry probe were obtained using an analytical/precision weighing balance (0.001 g). A sterile 0.5 mL dry tube was assigned to each cut bELF probe. This tube was first weighed to obtain the weight of the empty tube. The bELF probe was then added to its assigned pre-weighed dry tube (0.5 mL), and the tube was weighed again for the weight of the dry probe. The bELF probes were stored and transported in their assigned tube at room temperature.

### 2.3. Airway-tissue contact testing

All three absorptive technologies were tested with airway tissue contact and evaluated on the adhesion to the surface and material strength. Freshly harvested porcine lungs were purchased from a commercial vendor (Kroon Vlees, Groningen, Netherlands). Probes cut from Whatman® Glass microfiber filters (Grade GF/C), Whatman® qualitative filter paper (Grade 3), and Whatman® qualitative filter paper (Grade *CF*-12) were applied onto porcine airway tissue. Probes from each matrix were gently pressed against the surface of the freshly harvested wet porcine airway for 30 s. After the removal of the probe, the contact surface of porcine airway tissue was imaged with a camera and evaluated for the deposition of any residual material.

### 2.4. Absorption capacity and elution efficiency testing

Testing for protein analyte collection and elution capacity was only carried out on the Whatman® qualitative filter paper (Grade *CF*-12) matrix. Two serial dilution series (20, 10, 5, 1 ng/mL) were prepared from a recombinant standard of human interleukin-8 (CXCL8) and human C-reactive protein (CRP; R&D Systems, Minnesota, United States). A sample of each solution from both series was collected to measure the original analyte concentration. For the experimental setup, 40 bELF probes stored in pre-weighed assigned 0.5 mL tubes were used. Two sets of four 1 mL aliquots containing 20, 10, 5, and 1 ng/mL of CXCL8 and CRP were prepared. Five bELF probes were designated for each of the eight solutions. One at a time, a bELF probe was removed from its assigned tube using a clean stainless steel tweezer and submerged for 10 s in its designated solution. Five bELF probe samples were collected from each of the eight serial dilution solutions.

### 2.5. Primary bELF probe sample processing and dilution-correction method

**Figure 2:**
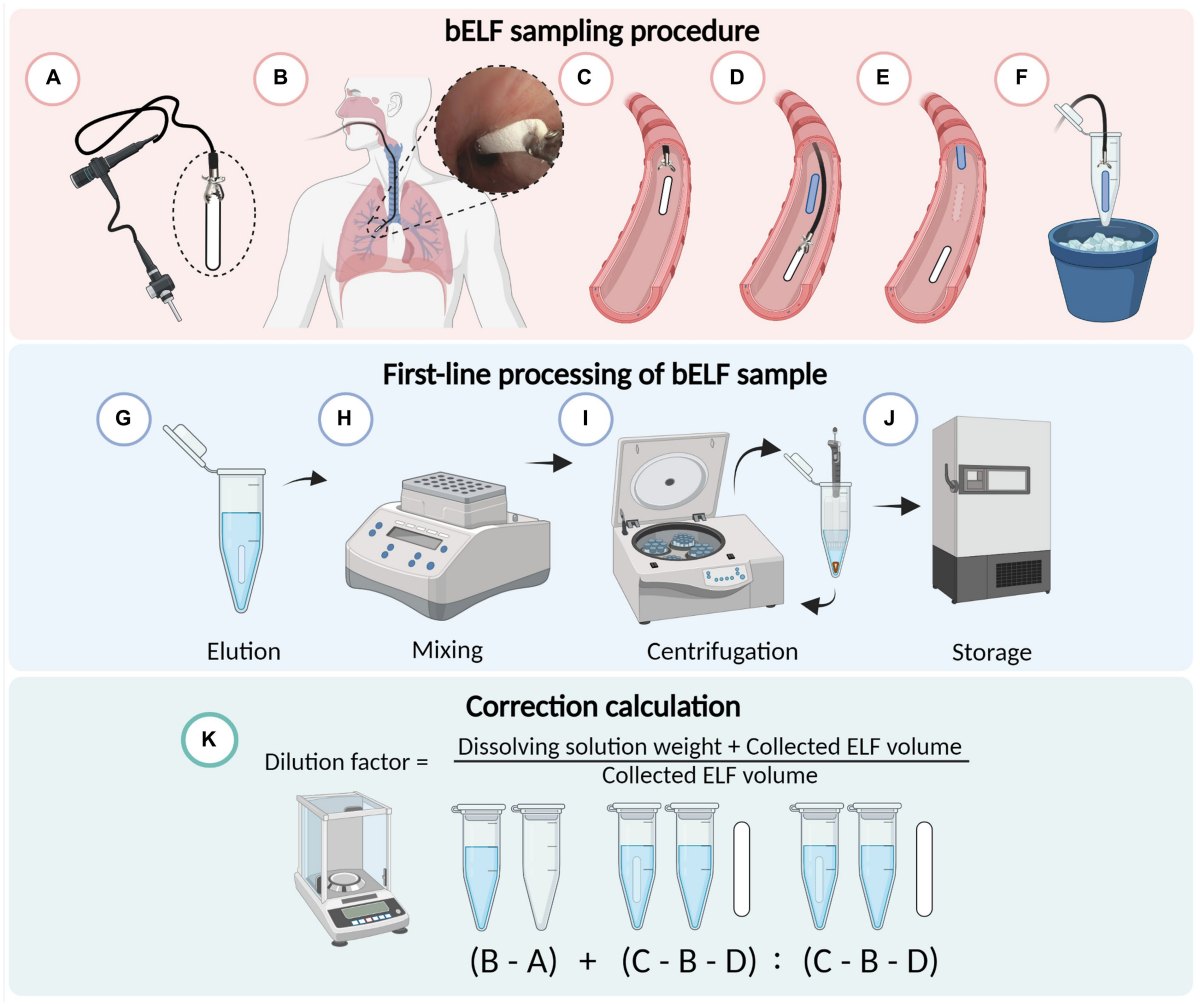
Schematic illustrating the bronchial epithelial lining fluid (bELF) probe sampling and laboratory processing protocol. Illustration of the minimally-invasive bronchoscopic bELF sampling procedure **(A–F)**, grasping the bELF probe with a biopsy forceps **(A)**, inserting the forceps and bELF probe through the bronchoscope working channel to the target site **(B)**, gently applying on the airway wall and releasing the bELF probe **(C)**, retracting the biopsy forceps to repeat the sampling protocol with a second bELF probe while waiting for absorption of the first probe **(D)**, removal of the saturated bELF probe **(E)**, storage of bELF probe after sampling on ice **(F)**. Illustration of the bELF probe laboratory processing method **(G–J)**. Submersion of bELF probe in elution buffer **(G)**, elution of collected proteins through shaking in a thermomixer **(H)**, separation of cellular debris from the eluted solution through centrifugation **(I)**, storage of supernatant at −80°C **(J)**. A calculation to correct measurements for dilution **(K)** using the weight of an empty tube **(A)**, tube with elution buffer **(B)**, tube with elution buffer and wet bELF probe **(C)**, and dry bELF probe **(D)**. Figure made in BioRender.com

Immediately after sampling, the wet bELF probe was directly returned to its assigned tube and reweighed for the weight of the collected fluid (Figures 2A-F). Subsequently, 300 μL of elution buffer (0.05%) Tween-20 (P1379) and 1% BSA (A2153; Sigma-Aldrich, Missouri, United States) in PBS was added to the tube containing the wet probe sample (Figure 2G). The tube was then reweighed for the weight of the elution buffer. Next, the sample tubes were shaken in a thermomixer (Eppendorf®, Hamburg, Germany) for 10 min at 600 rotations per minute (RPM) at room temperature (RT) to elute the collected proteins from the wet probe into the elution buffer (Figure 2H). The probe was then removed from the tube using clean stainless steel tweezers. To separate debris from the eluted fluid, the tube was centrifuged for 5 min at 600 g RT, and 250 μL of supernatant was aliquoted into a separate tube and stored at −80°C for further analysis (Figures 2I,J). A dilution factor was calculated for each bELF probe sample using the weight measurements of its assigned empty tube, the dry probe, the wet probe, and the weight of the added elution buffer. For the calculation, it was assumed that the density of the elution buffer and collected bELF was one and that the same concentration of solutes was removed with the wet probe following elution, as remained in the eluted sample (Figure 2K) (11).

### 2.6. Bronchoscopy procedure and sample collection

The bELF probe and sampling method was tested in 10 patients with severe COPD who were scheduled for a bronchoscopic lung volume reduction procedure under general anesthesia and participated in the “Biological Investigation of Explanted Endobronchial Lung Valves” Study (Bio-EXCEL study, NCT04214587; Group 1; Table 1). The bronchosorption device samples were obtained later from a separate group of 6 current or ex-smokers (Group 2). These patients participated in the “An Integrative Genomic Approach to Solve tHe Puzzle of sevERe earLy-Onset COPD” study (SHERLOCk study, NCT04263961) and were scheduled for a study-related bronchoscopic sampling under general anesthesia (Table 1). Both studies were approved by the UMC-Groningen ethics committee, and all patients provided written informed consent.

**Table 1:** Patient characteristics.

	Group 1	Group 2
Age, years Median (range)	65 (55–71)	64 (54–69)
Sex, *n* (male/female)	4/6	4/2
FEV1, %predicted Mean (SD)	26.1 (5.7)	73.3 (42.3)

FEV1, Forced Expiratory Volume; SD, Standard Deviation.

Bronchial epithelial lining fluid was collected from a target subsegmental bronchus using the bELF probes and bronchial wash technique. Bronchial epithelial lining fluid was first collected using three bELF probes. The bELF probe was grasped at the short end with biopsy forceps outside the bronchoscope and advanced through the endotracheal tube in front of the bronchoscope to the desired position and gently released on the bronchial wall for the absorption of ELF for an absorption time of 30 s for full saturation of the absorptive matrix. The primary fully-saturated probe was then grasped back with the biopsy forceps and retracted, still in front of the bronchoscope, through the endotracheal tube. Following extraction of the wet probe, the probe was directly returned to its assigned tube and placed on ice. For multiple samples from the same site, the procedure was simply repeated (Figures 2C–E).

After sampling was completed with the bELF probes, a bronchial wash was performed through injection of 20 mL of 0.9% saline solution, followed by immediate recovery. Following the collection of all samples, they were transported to the laboratory on ice (bELF probe, bronchial wash) for further processing within 2 h.

### 2.7. Primary bELF probe and bronchial wash processing

The collected bELF probes were processed and stored according to the primary processing method detailed above, and a dilution factor was calculated. The bronchial wash was centrifuged at 500 g at 4°C for 10 min, and supernatants were aliquoted. The collected supernatants were stored at −80°C for further analysis.

### 2.8. Bronchoscopy sampling and primary processing with bronchosorption device

Bronchial epithelial lining fluid was collected from patient group 2 using the bronchosorption catheter system (Mucosal Diagnostics, Midhurst, United Kingdom). One sample was collected from each patient. The sampling and primary processing method was carried out according to the protocol described by Thwaitse et al., and aliquoted supernatants were stored at −80°C for further analysis (12).

### 2.9. Measurement of analytes

Levels of CXCL8 (DY20), CRP (DY1707), or MPO (DY3174) were measured in duplicate using R&D systems human enzyme-linked immunosorbent assays (R&D Systems, Minnesota, United States) according to the manufacturer’s instructions. Absorbance was measured using the EL808 multi-well microplate reader, and the data was analyzed using Gen5™ data analysis software (Biotek® Instruments, Vermont, United States).

### 2.10. Statistical analysis

Validation data and patient data were characterized descriptively. Descriptive statistics, including mean, median, and 95% confidence intervals, were used to represent the variables of bELF, bronchial wash, and bronchosorption cytokine concentrations. A Mann–Whitney *U*-test was also performed. The points of sample measurements, above or below the limit of detection, were not included in the statistical analysis but were manually plotted for visual representation. All data were assessed and statistically analyzed using GraphPad Prism version 9.5.1 (GraphPad Software, California, United States). A value of *p ≤* 0.05 was considered significant.

## 3. Results

### 3.1. Airway-tissue contact testing

**Figure 3:**
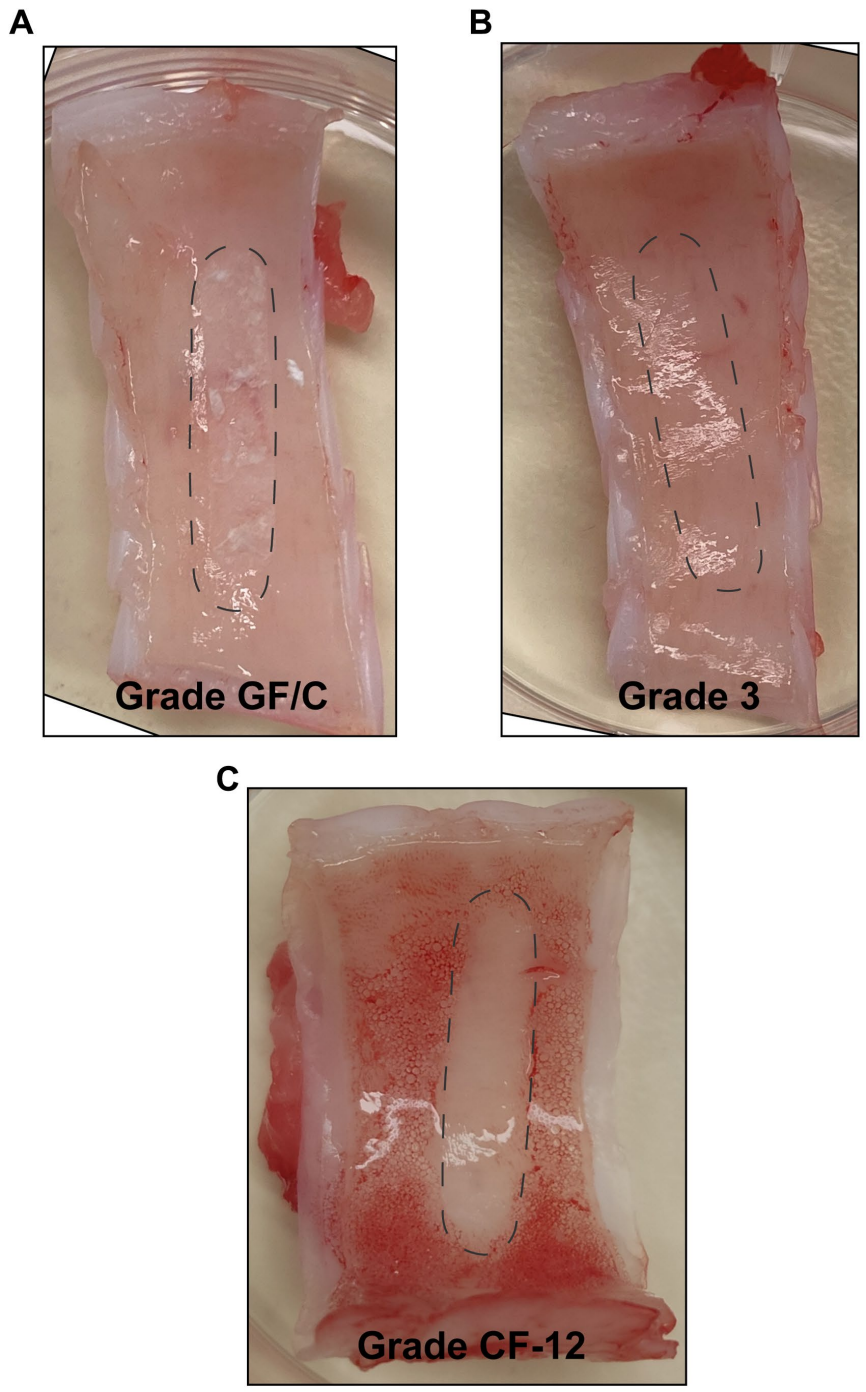
Assessment of structural integrity of absorptive matrices through porcine airway-tissue contact. Figures showing the surface of freshly harvested porcine-airway tissue after 30 s of contact with a bELF probe cut from Whatman® Glass microfiber filters (Grade GF/C) **(A)**, Whatman® qualitative filter paper (Grade 3) **(B)**, and Whatman® qualitative filter paper (Grade *CF*-12) **(C)**. The position of the probe during contact is represented with dotted lines.

The adhesive properties and material strength of Whatman® filter papers Grade GF/C, Grade 3, and Grade *CF*-12 were evaluated through contact with airway tissue. All three filter papers adhered to the porcine airway tissue upon wetting. From observation, grade GF/C filter paper adhered the strongest and grade 3 the weakest. The Whatman® Glass microfiber filters (Grade GF/C) softened the most upon wetting, and residual fibers were consistently observed on the airway tissue following the removal of the probe, shown in Figure 3A. The Whatman® qualitative filter paper (Grade 3) remained rigid upon wetting through porcine airway contact, and the matrix remained structurally intact (Figure 3B). Whatman® filter paper Grade *CF*-12 softened slightly when wet but remained structurally intact, with no evidence of residual cellulose fibers remaining on the airway tissues (Figure 3C). Following airway tissue contact testing, the Whatman® Glass microfiber filters (Grade GF/C) and Whatman® qualitative filter paper (Grade 3) were deemed incompatible for bELF sampling and excluded from further testing.

**Figure 4:**
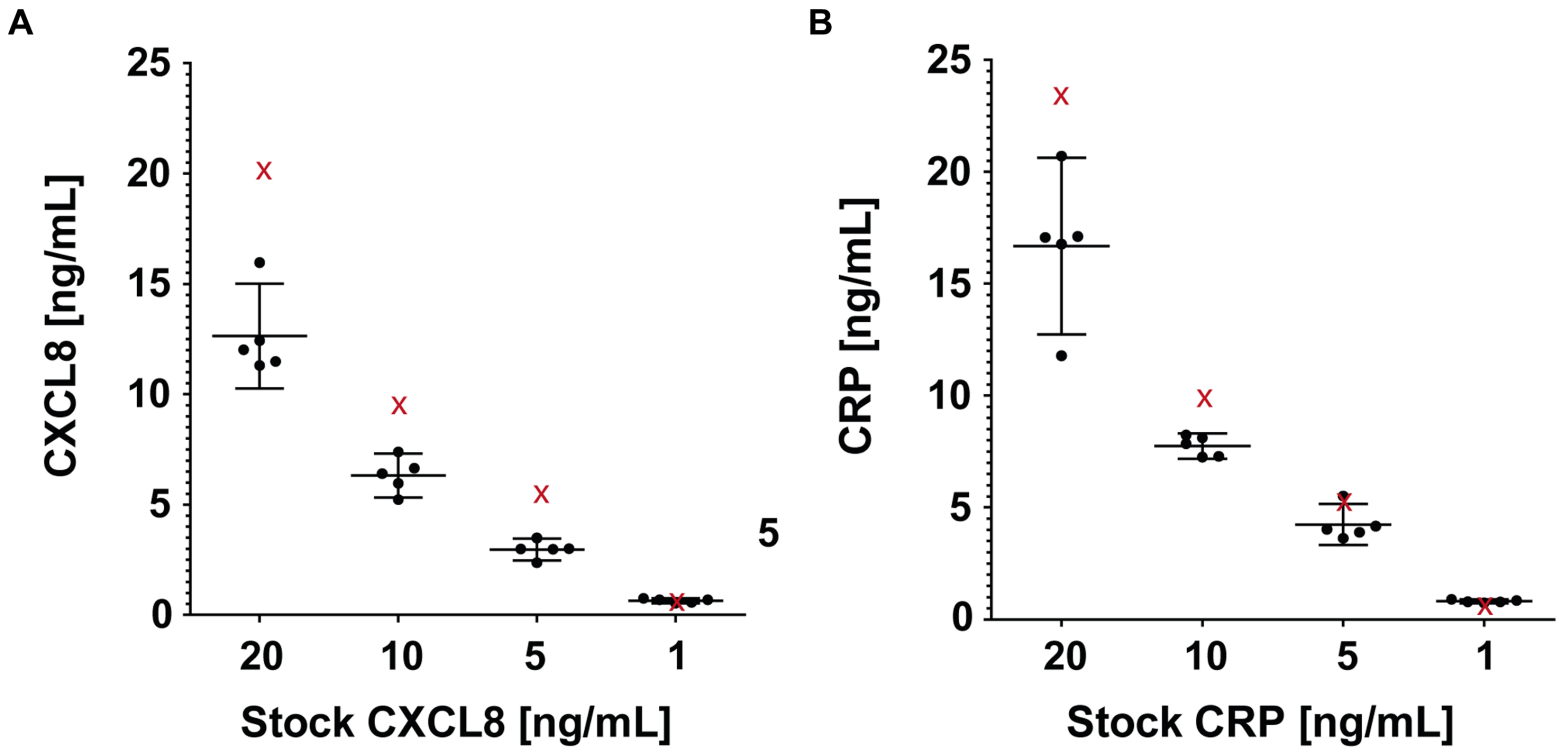
Characterization of the absorption capacity and elution efficiency of the bronchial epithelial lining fluid (bELF) probe. The graphs illustrate interleukin-8 (CXCL8) **(A)** and C-Reactive protein (CRP) **(B)** levels measured using enzyme-linked immunosorbent assays in samples collected using a bELF probe (Whatman® grade *CF*-12 filter paper) from a CXCL8 and CRP serial dilution series (20, 10, 5, 1 ng/mL). Represented for each solution in both series are the analyte measurements of a portion aliquoted for the original concentration (red cross) before bELF probe sampling and a set of five bELF probe samples, corrected for dilution (black dot). The bELF probe sample data are represented as mean (95% CI).

A total of 40 probes, produced from Whatman® filter paper Grade *CF*-12, were submerged in serial dilution series of CXCL8 and CRP (20, 10, 5, and 1 ng/mL; Figure 4). After 10 s of complete submersion, the bELF probes collected, on average, 32 μL (range 24−35 μL) of the solutions. The mean return of CXCL8 and CRP using the bELF probe sampling and processing method was 69% (range 48%–87%). The mean coefficient of variation for the bELF probe sample (*n* = 5) replicates measurements was 0.13 (Range: 0.06–0.19; Figure 4).

### 3.2. Bronchoscopy procedure

The bronchoscopic sampling method was feasible for a trained respiratory physician and was well tolerated by all patients. The mean amount of bronchial epithelial lining fluid collected by the bELF probes was 21 μL (Range 4−46 μL).

### 3.3. Biomarker analysis

**Figure 5:**
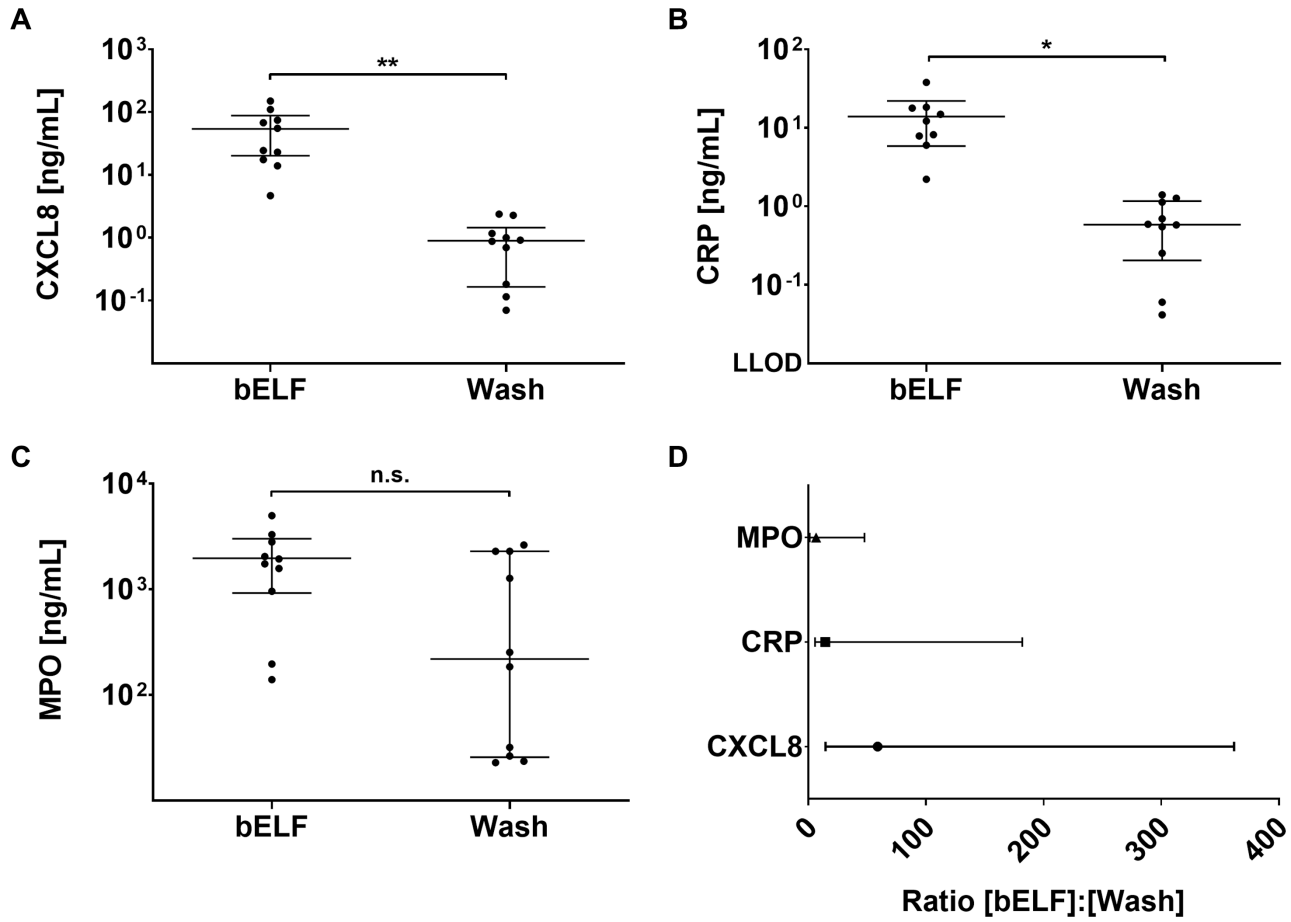
Levels of pro-inflammatory proteins obtained from the bronchial epithelial lining fluid (bELF) probe and bronchial wash of a COPD patient group *n* = 10. The graphs show the levels of CXCL8 **(A)**, C-Reactive protein (CRP) **(B)**, and Myeloperoxidase (MPO) **(C)** measured using enzyme-linked immunosorbent assays in bELF and bronchial wash collected from 10 COPD patients during routine bronchoscopy. The data are represented as a median (95% confidence interval) with a Mann–Whitney *U*-test. Non-significant (n.s.), *p* ≤ 0.05 (*), *p* ≤ 0.001(**), Not detected (N.D.) The ratio of pro-inflammatory protein concentrations obtained from the bELF probe and by bronchial wash **(D)**. The data are represented as median (IQR). The lower limit of detection (LLOD).

The concentrations of CXCL8, CRP, and MPO were measured in the supernatants of the bELF probe and bronchial wash samples collected from 10 COPD patients (Figures 5A–C). The measured concentrations of CXCL8 and CRP were higher in all epithelial lining fluid samples collected with the bELF probe compared to the sample collected with bronchial wash. For MPO, two patients had a higher concentration of MPO in the bronchial wash compared to the bELF probe measurement. The levels of CRP in the bELF samples of one patient from group 1 fell outside the assay dynamic range (Figure 5B). The measured protein concentration ratios of bELF: Wash were examined for each pair of samples. The median concentration of CXCL8 was 59 times higher (*p* < 0.0001) in bELF samples compared to bronchial wash and for CRP 14 times higher (*p* < 0.0001). The median concentration of MPO was 7 times higher, but there was no difference between the two groups (*p* = 0.0892; Figure 5D).

**Figure 6:**
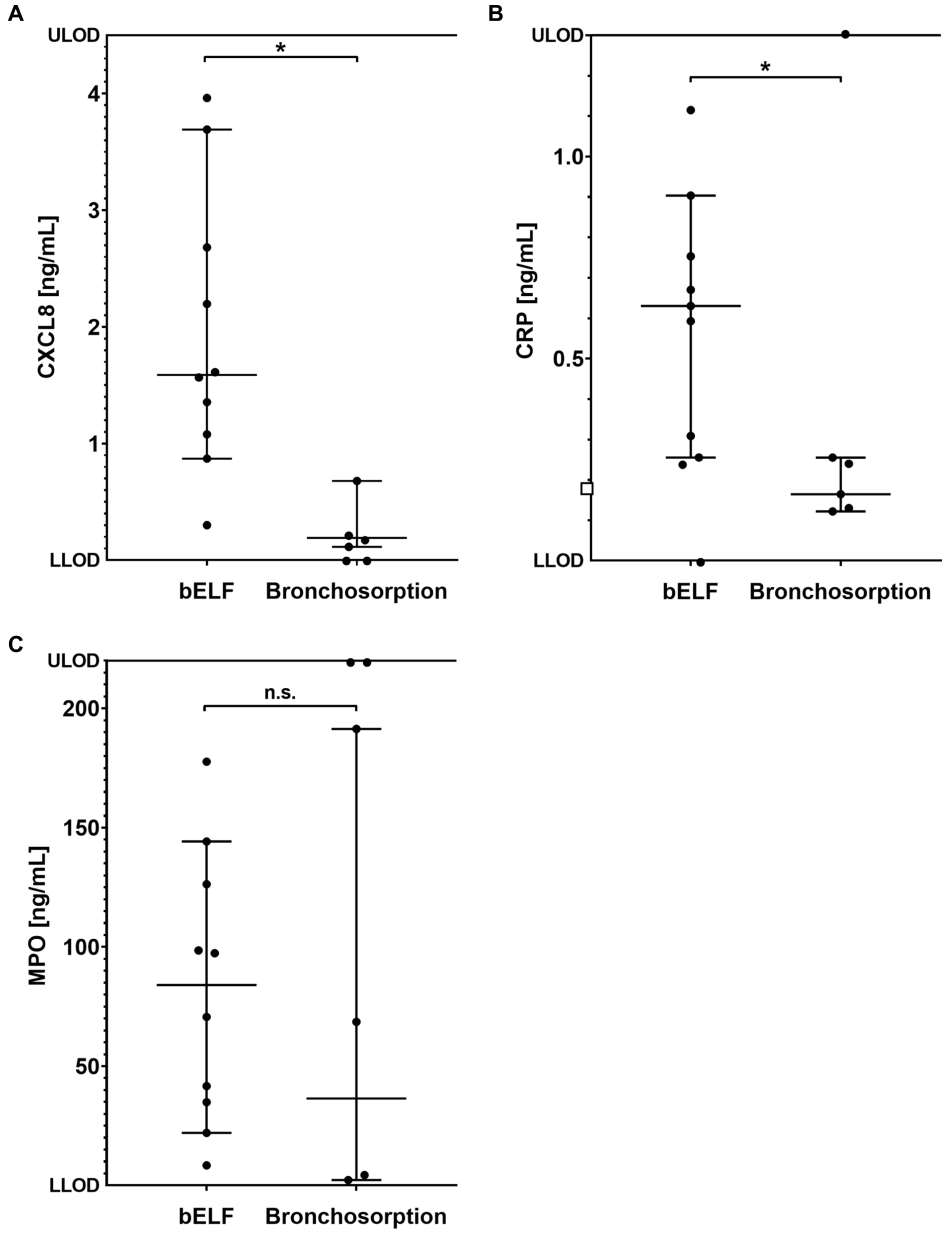
Levels of pro-inflammatory proteins obtained from bronchial epithelial lining fluid (bELF) sampled with the bronchosorption device (*n* = 6) and the bELF probe (*n* = 10). The graphs show the levels of CXCL8 **(A)**, C-Reactive protein (CRP) **(B)**, and Myeloperoxidase (MPO) **(C)** measured using enzyme-linked immunosorbent assays in samples of bELF collected with the bronchosorption device and the bELF probe from patients during routine bronchoscopy. LLOD, lower limit of detection; ULOD, Upper limit of detection.

The concentrations of CXCL8, CRP, and MPO were measured in the supernatants of the bronchosorption samples collected from 6 current and ex-smokers (Figures 6A–C). The levels of CXCL8 and MPO felt outside the assay dynamic range in the bronchosorption samples of two patients from group 2, and CRP levels felt outside for one (Figure 6). The median concentration of CXCL8 measured in the bELF samples was approximately 8 times higher than in the bronchosorption samples (*p* = 0.0040). The median concentration of CRP was approximately 4 times higher (*p* = 0.0040). The median concentration of MPO was 2 times higher, but there was no difference between the two groups (*p* = 0.4535).

## 4. Discussion

This study explored a novel cellulose-based absorptive matrix as a candidate for a respiratory epithelial lining fluid sampling device. The Whatman qualitative filter paper grade *CF*-12 was selected as the optimal absorptive matrix for the bELF sampling probe due to its maintenance of structural integrity during sampling and absorption and elution efficiency. The bELF probe production, sampling, processing, and correction method were developed to be universally applicable in any bronchoscopy center with basic laboratory equipment. The bELF probe was well-tolerated by patients and returned a higher yield for inflammatory biomarkers in bELF compared to the current standard bronchial wash.

The practice of sampling mucosal surfaces with filter papers is well-established and has been applied previously for the collection of nasal epithelial lining fluid, rectal mucosal fluid, and dental crevicular fluid (13–16). Several filter paper technologies have been explored in the past. In this study, after testing three distinct filter papers, Whatman™ grade *CF*-12 filter papers were found to be the most optimal candidate sampling matrix. The *CF*-12 filter paper technology was developed and optimized for protein analysis with Whatman™ 903 cards, a bio-sample (e.g., blood, urine) collection, and transport product. These filter papers are 100% pure cotton linter with no-wet strength additives. This material composition equipped the bELF probes with high absorption capacity and rapid wicking (17). These features facilitate the collection of bELF from a target site using multiple bELF probes while maintaining a short procedure time and with each bELF probe collecting sufficient volume for protein analysis with duplicate measurements. Additionally, the wettability and strength of the matrix determined the ease of use and safety of the sampling method. Contact bleeding is an important complication reported previously in sampling asthmatic airways with the BMS probe (18). Injury to the airway mucosa with the BMS probe could be attributed to the use of a hard absorptive matrix or the embedding of a metal wire through the filter paper tip for structural integrity and continuity with the catheter system. From our experience, with careful extrusion and gentle application of the bELF probe, contact bleeding and contamination of the sample could be prevented.

Arguably, the most important property of a sampling matrix is its efficiency in the absorption and elution of analytes. During our controlled experimental setup, the bELF probe sampling and processing method returned, on average, 69% (range 48%–87%) from the serial dilution series of CXCL8 and CRP. The low variability between the replicate measurements highlights the high precision of the measurements. To improve the returned yield, non-specific binding to the inner surfaces of the sample tubes could be reduced by using specific low-protein binding tubes or pre-coating the tubes with a protein matrix, such as albumin. Additionally, to improve protein extraction, parameters of the elution buffer, such as pH and salt content, could be adjusted. Likewise, the time, temperature, and speed of shaking with the thermomixer could be optimized. Lastly, an electrochemiluminescence-based immunoassay could have been employed to improve the sensitivity of the analysis.

The dilution factor calculation was critical to maximize the accuracy of quantified values. Under our controlled experimental setup dipping 40 bELF probes until complete saturation, a relatively small range of dilution factors (13 to 21) was calculated. Dilution factors calculated from bELF samples collected from the 10 COPD patients ranged from 8 to 78. The small range of dilution factors during the optimization experiments is likely due to a consistent dipping method till full saturation and collection of a uniform PBS-based protein stock solution. The viscosity of bELF remains a challenge for all respiratory sampling methods and was also encountered with the bELF probe when sampling severe COPD respiratory tracts layered intermittently with highly viscous bELF. When samples collected from the same airway segment vary in collected bELF viscosity, the concentrations of suspended analytes may also vary, leading to variability in the quantification of analytes and greater variation across samples from the same segment.

The bELF probe significantly improved spatial informativity and sensitivity in measuring inflammatory biomarkers compared to bronchial wash. The bELF probe samples are obtained from the specifics section of the airway with which the device is in contact. This level of spatial informativity is most relevant for biomarker profiling in heterogenous airway disease, where lavage methods are limited in resolution. For instance, the bELF probe could be used to profile tissue reactions at the implant sites of airway devices (19). Furthermore, quantified levels of inflammatory biomarkers were, on average, multiple times higher with the bELF probe than those measured with bronchial wash. For CXCL8, for example, the bELF samples were measured to be 59 times higher than the bronchial wash obtained from the same segment. This difference introduces the potential for the quantification of low-concentration suspended analytes. The bELF samples collected with the probe in our study were not in contact with blood during the bELF sampling procedure, as sampling was performed with direct visual observation. On the other hand, the bronchial wash may have been contaminated as it was collected from sites, not under direct observation. Blood contamination can significantly alter the measurement of corresponding mediators as analytes may be several magnitudes higher in concentration in blood. Additionally, there is currently no standardized method for correcting for dilution in the bronchial wash (7).

Quantified levels of CXCL8, CRP, and MPO were, on average, higher in the bELF samples measured with the bELF probe than those measured with the commercially-available bronchosorption system. First, it is important to highlight this comparison’s limitations, which include the comparison of inflammatory biomarker concentrations of two different patient groups and the falling of several measurements from the limited sample outside of the ELISA dynamic range. Nevertheless, the data obtained shows that the bELF probe is a comparable absorptive matrix for measuring inflammatory biomarkers. Furthermore, the difference in concentrations may be attributed to the lack of dilution correction described in the bronchosorption method (12). Our data shows that, correction with a dilution factor is critical in the measurement of soluble proteins, as the magnitude is similar to the dilution factor of samples obtained with bronchial wash. This calculation was first described by Yamazi et al. for the measurement of drug concentrations with the BMS probe, and can easily be adopted into any bELF sampling method (11).

Guiding the bELF probe requires a degree of bronchoscopy handling competency. Transforming the bELF probe into a single-use catheter-based system, similar to the BMS and bronchosorption devices (9, 10), would improve the ease of use. However, this improvement in handling would provide a higher cost, limiting access for many centers worldwide. With the currently described method, the cost of producing individual bELF probes is nearly negligible. Additionally, open-source capacity would be abandoned through commercialization, limiting community-led improvements. On the other hand, developing a dedicated reusable catheter system for filter paper strips might be an option for further exploration.

The bELF probe is currently an effective alternative to bronchial wash for biomarker analysis, but future studies are necessary. Optimizing elution yields will require further investigation into the impact of viscosity, the type of biomarkers, and elution buffer constituents. Exploration of new applications, such as microbiological and proteomics analysis, will require validation. Additionally, testing for batch variation and implementation in multiple centers is needed to evaluate the replicability of this method.

Overall, the bELF probe is a simple, safe, and effective bronchoscopy-guided technique to generate samples for analyzing biomarkers in bELF from the lower respiratory tract. The bELF probe equips physicians with a reliable diagnostic, prognostic, and monitoring tool for various airway diseases by improving the precision and accuracy of obtained bELF biomarker measurements.

## Data availability statement

The raw data supporting the conclusions of this article will be made available by the authors, without undue reservation.

## Ethics statement

The studies involving human participants were reviewed and approved by Medical Ethics Review Board University Medical Center Groningen. The patients/participants provided their written informed consent to participate in this study.

## Author contributions

AG, JB, D-JS, and SP were involved in the conception and design of the study, were involved in interpretation of the generated data, and were involved in revising the manuscript. AG performed the experimental work, analyzed the data, generated figures, and was involved in drafting the manuscript. D-JS contributed by performing the bronchoscopic sampling. All authors contributed to the article and approved the submitted version.

## Funding

This project was co-financed by the Ministry of Economic Affairs and Climate Policy by means of the PPP-allowance made available by the Top Sector Life Sciences and Health to stimulate public-private partnerships, with project code PPP2019-043.

## Conflict of interest

The authors declare that the research was conducted in the absence of any commercial or financial relationships that could be construed as a potential conflict of interest.

## Publisher’s note

All claims expressed in this article are solely those of the authors and do not necessarily represent those of their affiliated organizations, or those of the publisher, the editors and the reviewers. Any product that may be evaluated in this article, or claim that may be made by its manufacturer, is not guaranteed or endorsed by the publisher.
